# TrkB is highly expressed in NSCLC and mediates BDNF-induced the activation of Pyk2 signaling and the invasion of A549 cells

**DOI:** 10.1186/1471-2407-10-43

**Published:** 2010-02-16

**Authors:** Siyang Zhang, Dawei Guo, Wenting Luo, Qingfu Zhang, Ying Zhang, Chunyan Li, Yao Lu, Zeshi Cui, Xueshan Qiu

**Affiliations:** 1 Center of Laboratory Technology and Experimental Medicine, China Medical University, Shenyang, China; 2 Department of General Surgery, the Fourth Affiliated Hospital of China Medical University, Shenyang, China; 3 Department of Pathology, the First Affiliated Hospital and College of Basic Medical Sciences of China Medical University, Shenyang, China

## Abstract

**Background:**

Aberrant regulation in the invasion of cancer cells is closely associated with their metastatic potentials. TrkB functions as a receptor tyrosine kinase and is considered to facilitate tumor metastasis. Pyk2 is a non-receptor tyrosine kinase and integrates signals in cell invasion. However, little is known about the expression of TrkB in NSCLC and whether Pyk2 is involved in TrkB-mediated invasion of A549 cells.

**Methods:**

The expression of TrkB was investigated in NSCLC by immunohistochemical staining. Both HBE and A549 cells were treated with BDNF. The expression of TrkB, Pyk2 and ERK phosphorylations were assessed by western blot. Besides, A549 cells were transfected with TrkB-siRNA or Pyk2-siRNA, or treated with ERK inhibitor where indicated. Transwell assay was performed to evaluate cell invasion.

**Results:**

40 cases (66.7%) of NSCLC were found higher expression of TrkB and patients with more TrkB expression had significant metastatic lymph nodes (p = 0.028). BDNF facilitated the invasion of A549 cells and the activations of Pyk2 in Tyr402 and ERK. However, the effects of BDNF were not observed in HBE cells with lower expression of TrkB. In addition, the increased Pyk2 and ERK activities induced by BDNF were significantly inhibited by blocking TrkB expression, so was the invasion of A549 cells. Knockdown studies revealed the essential role of Pyk2 for BDNF-induced cell invasion, since the invasion of A549 cells was abolished by Pyk2-siRNA. The application of ERK inhibitor also showed the suppressed ERK phosphorylation and cell invasion.

**Conclusion:**

These data indicated that higher expression of TrkB in NSCLC was closely correlated with lymph node metastasis, and BDNF probably via TrkB/Pyk2/ERK promoted the invasion of A549 cells.

## Background

Lung cancer is the leading cause of death among the malignant tumors worldwide, and the incidence of non-small cell lung cancer (NSCLC) is increasing. The prognosis of patients with NSCLC principally correlates with tumor metastasis, which involves the regulation of some critical genes and more information should be gathered on the research of those prometastatic genes.

Tropomysin-related kinase B (TrkB) is a member of Trk family, functions as a receptor tyrosine kinase. Brain-derived neurotrophic factor (BDNF), the primary ligand, binding to TrkB results in the regulation of various cellular activities in neuroblastoma, such as cell differentiation [1], apoptosis [2], and invasion [3]. TrkB is up-regulated in a variety of primary human tumors, including neuroblastoma [4] and ovarian cancer [5], especially in metastatic gastric [6] and pancreatic tumors [7]. Enhanced TrkB signaling promotes cell survival in an anchorage-independent manner [8]. When activated by BDNF, TrkB leads to the activation of downstream signaling molecules, such as phosphoinositide-3 kinase/protein kinase B (PI3K/Akt) [9-11], which induces the differential regulation of apoptosis and metastasis. However despite the increasing emphasis on TrkB in human tumors, whether it positively participates in primary human NSCLC has not yet been determined. At present, little is known about the molecular mechanisms that elicit signalings downstream of TrkB in the progression of NSCLC.

Proline-rich tyrosine kinase 2 (Pyk2) is an extensively expressed non-receptor tyrosine kinase and integrates signals from receptor tyrosine kinases and intracellular signaling molecules in the essential cellular processes such as cell differentiation [12], proliferation [13] and migration [14]. Pyk2 is rapidly tyrosine phosphorylated in response to various extracellular signals [15,16] and activated Pyk2 signaling promotes cell survival and migration in an anchorage-independent manner [17]. The tyrosine 402 (Tyr402) of Pyk2 serves as the primary autophosphorylation site that is essential for Pyk2 activity and function [18], which is supported by the high activity of Tyr402 found in tumor cells with a more invasive and metastatic phenotype [19,20].

This study is designed to investigate the expression and clinical significance of TrkB in 60 cases of surgically resected NSCLC and the potential downstream signaling of TrkB in BDNF-induced invasion of A549 cells. We reported here that high expression of TrkB was common in NSCLC, particularly correlated with lymph node metastasis and TNM stage. We also reported that TrkB-siRNA interrupted BDNF-promoted Pyk2 and extracellular regulating kinase (ERK) activations and invasion of A549 cells. Similarly, Pyk2-siRNA inhibited BDNF-associated ERK phosphorylation and cells invasion. Therefore, TrkB/Pyk2/ERK signaling was considered to mediate BDNF-induced invasion of A549 cells. These results identify TrkB as a potential novel regulator of cell invasion and the suppression of TrkB may provide a helpful target for inhibitory therapies of metastasis in NSCLC.

## Methods

### NSCLC Samples

A total of 60 cases of NSCLC were obtained from the Pathology Department of China Medical University. This study was approved by the Medical Research Ethics Committee of China Medical University and the informed consent was obtained from all patients. All of the enrolled patients underwent curative surgical resection without having chemotherapy or radiation therapy. Formalin-fixed paraffin-embedded sections of tumor were stained routinely with hematoxylin and eosin (HE), and reviewed by two senior pathologists in order to determine the histological type and stage, according to the WHO classification of lung and pleural tumors (2004) and the TNM staging system (1997). Lymph node status was determined by routine pathological examination of dissected nodes. Clinicopathological information of the patients about tumor size, histological type, differentiation, stage and lymph node metastasis was obtained from patient records, and summarized in Table 1.

**Table 1:** Clinicopathological characteristics of 60 cases of NSCLC and TrkB expression by immunohistochemistry.

Clinicopathological charateristics	Cases (n = 60)	Higher exression (n = 40)	Lower expression (n = 20)	P value
Tumor size and invasiveness				
T1+T2	40	27	13	0.846
T3+T4	20	13	7	
Histological type				
Sq	25	15	10	0.355
Ad	35	25	10	
Differentiation				
well-moderate	44	28	16	0.409
Poor	16	12	4	
Stage				
I+II	34	19	15	0.043*
III	26	21	5	
Lymph node status				
+	30	24	6	0.028**
-	30	16	14	

TrkB expression: * = significant difference between early (I+II) and advanced (III) stage of NSCLCs; ** = significant difference between tumors with positive (+) and negative (-) nodes.

### Immunohistochemistry

60 paraffin sections of NSCLC were deparaffinized and rehydrated routinely. The recovery of antigens was performed by heating the slides in an autoclave sterilizer for 2 min in 0.1 mol/L Tris-HCl at pH10. The sections were incubated overnight at 4°C with primary rabbit polyclonal antibody detecting TrkB (1:100 dilution, Santa Cruz), following 3% H_2_O_2 _and 5% rabbit serum treatment at 37°C for 1 h. After which they were incubated with second antibody and streptavidin-peroxidase (SP) complex for 30 min (SP kit, MaiXin, China), and then visualized with 3,3'-diaminobenzidine (DAB). Neuroblastoma sections were used as positive controls for TrkB, and negative controls were prepared by non-immune rabbit IgG. All the immunoreactions were separately evaluated by two senior pathologists. Cells with brown particles appearing in cell membrane or cytoplasm was as regarded as TrkB-positive. The intensity of TrkB immunostaining (1 = weak, 2 = intense) and the percentage of positive tumor cells (0% = negative, 1-50% = 1, 51-75% = 2, ≥ 76% = 3) were assessed in at least 5 high power fields (×400 magnification). The scores of each tumorous sample were multiplied to give a final score of 0, 1, 2, 3, 4, or 6, and the tumors were finally determined as negative: score 0; lower expression: score ≤ 3; or higher expression: score > 3.

### Cells culture and treatments

Human bronchial epithelial (HBE) and lung adenocarcinoma A549 cells were preserved in our department. HBE cells were grown in RPMI 1640 and A549 cells were cultured in DMEM (Invitrogen) supplemented with 10% fetal bovine serum (FBS), 100 U/ml penicillin and streptomycin, in incubator with 5% CO_2 _at 37°C. HBE and A549 cells (80-90% confluence) were firstly treated with 100 ng/ml BDNF for 24 h. To knockdown TrkB or Pyk2 for subsequent studies, A549 cells (50-60% confluence) were transfected with either TrkB- or Pyk2-siRNA and scrambled control siRNA (GeneChem, China) for 48 h using Lipofectamin2000 (Invitrogen), according to the manufacturer's instructions. Cells were then treated with 100 ng/ml BDNF at 24 h after transfection and maintained for another 24 h. Where indicated, the ERK inhibitor (PD98059, Calbiochem) at 100 μmol/L was added to cells for 48 h. Cells were also treated with 100 ng/ml BDNF at 24 h after PD98059 treatment and maintained throughout the experiments. Those cells treated were used for proteins extraction or cell invasion analysis as described below. The experiments for cells were repeated at least three times.

### Cell invasion analysis

Cell invasion assay was performed using a 24-well Transwell chamber (Costar). At 24 h following treatments as described above, cells (1 × 10^4^) were detached and seeded in the upper chamber (containing 100 ng/ml BDNF) with an 8 μm pore size insert precoated with Matrigel (BD Biosciences) in the 24-well plate and cultured for another 24 h. Cells were allowed to migrate towards medium containing 15% FBS in the bottom chamber. The non-migratory cells on the upper membrane surface were removed with a cotton tip, and the migratory cells attached to the lower membrane surface were fixed with 4% paraformaldehyde and stained with hematoxylin. The number of migrated cells was counted in 5 randomly selected 200× power fields under microscope. Data expressed are representative of three individual wells.

### Western blot

Cells were washed twice with ice-cold phosphate buffer saline (PBS) and lysed in lysis buffer containing 20 mM Tris-HCl, 1 mM EDTA, 50 mM NaCl, 50 mM NaF, 1 mM Na_3_VO_4_, 1% Triton-X100, 1 mM phenylmethyl sulfonylfluoride (PMSF) and phosphatase inhibitor. The homogenate was centrifuged at 15000 rpm for 30 min at 4°C. The supernatant was extracted and protein content was determined by the BCA (bicinchoninic acid) assay (Pierce). 80 μg of total protein was separated by sodium dodecyl sulfate-polyacrylamide gel electrophoresis (SDS-PAGE) and then transferred to polyvinylidene fluoride (PVDF) membrane. After blocking with 5% bovine serum albumin (BSA), primary antibodies including rabbit polyclonal anti-TrkB, anti-Pyk2, anti-p-Tyr402, anti-β-actin, mouse monoclonal anti-p-ERK (all from Santa Cruz) were incubated on the membranes overnight at 4°C. The membranes were then incubated for 2 h at 37°C with secondary antibodies (ZhongShan, China). Immunoreactive straps were identified using the enhanced chemiluminescence (ECL) system (KeyGEN, China), as directed by the manufacturer. The DNR Imaging System was used to catch up the specific bands, and the optical density of each band was measured using the Image J software. The ratio between the optical density of interest proteins and β-actin of the same sample was calculated as the relative content of protein detected.

### Statistical analysis

The SPSS 13.0 software was applied to complete data processing. χ^2^-test was applied to analyze the correlations between TrkB expression and clinicopathological characteristics. T-test or One-way ANOVA was used to compare the differences between cells with various treatments. All data were represented as mean ± SD and results were considered statistically significant when the p-value was less than 0.05.

## Results

### The Expression of TrkB in 60 NSCLCs by Immunohistochemistry

**Figure 1:**
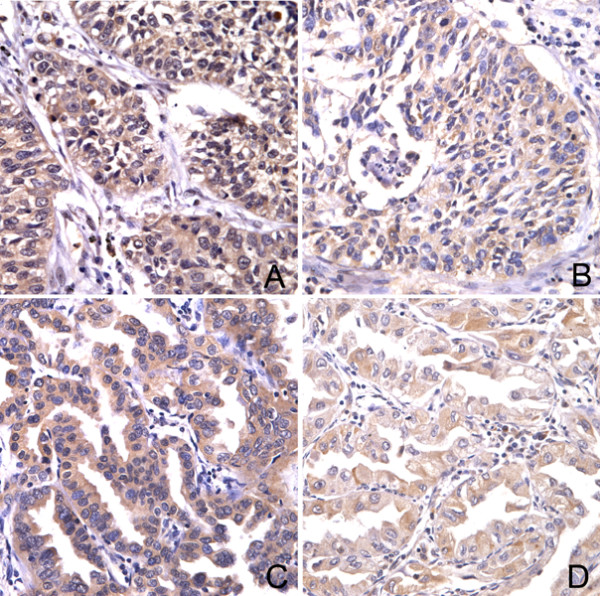
**The expression of TrkB in NSCLC examined by immunohistochemistry**. TrkB immunostaining in lung squamous cell carcinoma with (A) and without (B) lymph node metastasis. TrkB immunoreactivity in lung adenocarcinoma with (C) and without (D) positive nodes. It was suggested that TrkB expression was correlated with lymph node status. Original magnification, all ×400.

TrkB immunoreactivity was detected in 52 (86.7%) neoplastic sections. We considered that 40 (66.7%) cases of NSCLC were higher expression (scores of 4 or 6) and 20 cases (33.3%) were lower expression (scores of 0, 1, 2 or 3), as described above in Materials and methods. TrkB has been reported to facilitate tumor metastasis [21,22], and the association between TrkB expression and the presence of lymph node metastasis at the time of resection was analyzed statistically. TrkB immunostaining was stronger in NSCLCs with lymph node metastasis compared with those node negative cases and a statistically significant correlation between higher TrkB expression and positive node was found (P = 0.028). In addition, patients with higher TrkB expression had advanced stage of NSCLC (I+II versus III, P = 0.043). However, no significant difference of TrkB expression was found between tumor size (T1+T2 versus T3+T4, P = 0.846), histological type (Ad versus Sq, P = 0.355) and differentiation (well-moderate versus poor, P = 0.409). Samples of TrkB expression in NSCLCs with and without lymph node metastasis are shown in Figure 1. The correlations of TrkB expression and clinicopathological characteristics are shown in Table 1.

### Effect of BDNF on Cell Invasion

**Figure 2:**
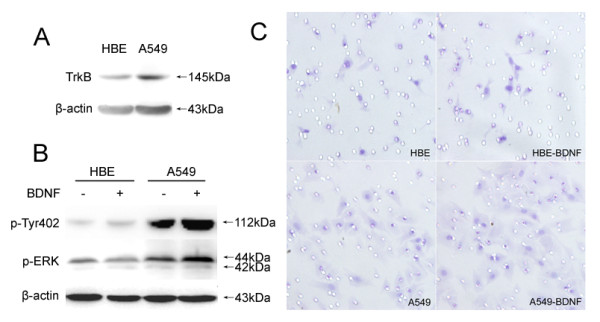
**Comparison of TrkB expression in HBE and A549 cells by western blot**. HBE cells exhibited a minimal amount of TrkB and much higher level of TrkB was found in A549 cells (A). In the presence of BDNF, the phosphorylations of Pyk2 in Tyr402 and ERK were promoted in TrkB-positive A549 cells, which were not observed in TrkB-null HBE cells (A). Simultaneously, the invasive number of A549 cells was increased after BDNF treatment, while the invasion of HBE cells was not affected (B). Original magnification, all ×400. The experiments for cells were repeated at least three times.

To investigate the potential signaling induced by BDNF that regulates cell invasion, HBE and A549 cells were used in this study. TrkB expression was examined in HBE and A549 cells and simultaneously, the invasion of these cells treated by BDNF was analyzed by Transwell assay. A549 cells exhibited much higher level of TrkB, which was hardly detectable in HBE cells (Figure 2A). As shown in Figure 2C, the invasive numbers of HBE and A549 cells with or without BDNF treatment at 24 h time point were 10.8 ± 1.4, 11.7 ± 1.9 (p = 0.549) and 19.5 ± 3.5, 30.7 ± 5.0 (P = 0.033), respectively. We also examined the activations of Pyk2 and ERK after BDNF treatments. Pyk2 phosphorylation in Tyr402 was increased in A549 cells upon BDNF stimulation, which was not observed in TrkB-null expressed HBE cells. The activity of ERK was also elevated by BDNF in A549 cells, compared with HBE cells (Figure 2B). These results showed that BDNF promoted the invasion of TrkB-positive A549 cells probably via Pyk2 phosphorylation in Tyr402, and the activations of both Pyk2 and ERK were participated in BDNF-induced invasion of A549 cells.

### Interruption of BDNF-induced Cell Invasion by TrkB-siRNA

**Figure 3:**
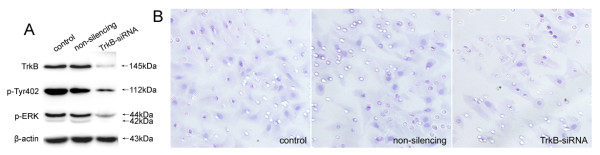
**Interruption of BDNF-induced cell invasion by TrkB-siRNA**. In TrkB-siRNA transfected A549 cells, the decreased activity of Pyk2 in Tyr402 promoted by BDNF was detected, which was much lower than that in non-silencing siRNA transfected and control cells. The activation of ERK was also attenuated after TrkB silencing (A). The invasion of TrkB-siRNA transfected A549 cells was greatly inhibited, in comparison to those non-silencing and control cells (B). Original magnification, all ×400. The data are representative of three individual experiments.

To define the role of TrkB in regulating BDNF-stimulated cell invasion, we utilized A549 cells due to the high expression of TrkB and the facilities of culture and transfection. The effects of decreased expression of TrkB on BDNF-induced cell invasion and the activations of Pyk2 and ERK by TrkB-siRNA were examined. Figure 3A showed that in TrkB-siRNA transfected A549 cells, the inhibited TrkB led to the decreased activity of Pyk2 elicited by BDNF, which was detected by the phosphorylation of Tyr402, lower than that in non-silencing siRNA transfected and control cells. The activation of ERK by BDNF was also attenuated after TrkB blocking. The invasive numbers of TrkB-siRNA, non-silencing and control A549 cells were 32.7 ± 2.8, 30.7 ± 4.3 and 18.6 ± 2.2 respectively (P = 0.003, Figure 3B). Therefore, TrkB knockdown cells exhibited reduced Pyk2 and ERK activations and diminished cell invasion.

### Effect of Pyk2-siRNA on BDNF-induced Cell Invasion

**Figure 4:**
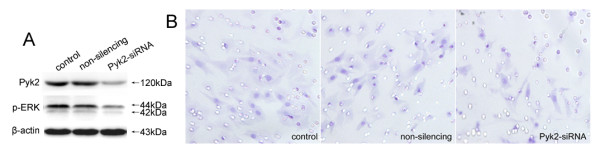
**Effects of Pyk2-siRNA on BDNF-induced cell invasion**. Pyk2 knockdown cells had TrkB expression unaffected, while the activation of ERK promoted by BDNF treatment was largely reduced (A). In addition, the invasive number of Pyk2-siRNA transfected A549 cells was significantly decreased and less than those non-silencing and control cells (B). Original magnification, all ×400. The data are representative of three replicates.

Studies have shown that Pyk2 mediated cell invasion [23], and we have observed the activation of Pyk2 by BDNF. We next transiently established Pyk2 knockdown A549 cells by specific siRNA to determine the role of Pyk2 in BDNF-induced cell invasion. Pyk2 knockdown cells had TrkB expression similar to those non-silencing and control cells, as shown in Figure 4A. However the activation of ERK by BDNF treatment was greatly reduced. In addition, the invasive numbers of Pyk2-siRNA, non-silencing and control A549 cells were 29.4 ± 3.8, 30.0 ± 3.0 and 16.9 ± 3.2, respectively (P = 0.005, Figure 4B). It seems that the inhibited expression of Pyk2 in those cells significantly correlated with decreased ERK activity and suppressed cell invasion induced by BDNF.

### Suppression of BDNF-induced Cell Invasion by PD98059

**Figure 5:**
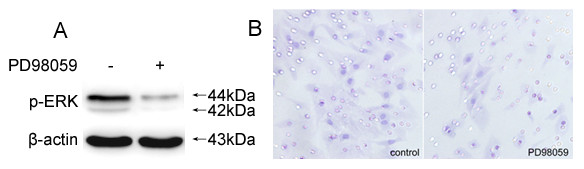
**Suppression of BDNF-induced cell invasion by PD98059**. The inhibited phosphorylation of ERK was observed after PD98059 treatment (A). The invasion of PD98059 treated A549 cells was diminished accordingly, compared with those non-silencing and control cells (B). It was indicated that the activated ERK was essential for regulating the invasion of A549 cells induced by BDNF. Original magnification, all ×400. The results are representative of three independent experiments.

We further investigated whether the activated ERK was definitely involved in BDNF promoted cell invasion by treating A549 cells with PD98059, a specific inhibitor for ERK. The inhibited phosphorylation of ERK was available by PD98059, as Figure 5A demonstrated. The invasive numbers of PD98059 treated or untreated A549 cells at 24 h were 27.9 ± 4.5 and 18.2 ± 3.6, respectively (P = 0.042, Figure 5B). Consequently, in the presence of PD98059, the activation of ERK was diminished concurrently with decreased invasive cells. It seems that the activated ERK played an essential role in regulating the invasion of A549 cells induced by BDNF. Collectively, these data suggested a novel, functional role of BDNF via TrkB in activating Pyk2 and ERK and enhancing the invasion of A549 cells.

## Discussion

The invasion of tumor cells plays a critical role for a successful metastasis. In this study, we investigated a potential signaling that regulates the invasion of A549 cells. Our data suggested a novel signaling by which BDNF facilitates the invasion of A549 cells via TrkB/Pyk2/ERK pathway, which possibly contributes to the metastasis of those lung cancer cells. Our study indicated that TrkB plays a critical role in promoting the invasion of A549 cells, which is mediated by a mechanism closely associated with the activations of Pyk2 and ERK.

The expression of TrkB is up-regulated in a variety of human tumors, such as hepatoma, pancreatic ductal adenocarcinoma, Wilms' tumor, astrocytoma and glioblastoma [24-27]. This study evaluated TrkB expression to determine the clinical significance of TrkB for the advanced NSCLC. We examined 60 cases of NSCLC by means of immunohistochemistry and found a statistical evidence of TrkB higher expression in NSCLC, and patients with higher TrkB expression had a significant metastatic phenotype, supporting the potential role of TrkB in survival and metastasis of tumor cells [28,29]. Therefore, the higher expression of TrkB probably plays an important role in the progression of NSCLC.

To investigate the potential function in BDNF-induced cell invasion, TrkB expression was compared between HBE and A549 cells. We found that the expression of TrkB in HBE cells was much lower, and A549cells with higher expression of TrkB seemed to be more invasive. Thus, TrkB was considered to be involved in the invasion of A549 cells. Compared with A549 cells, BDNF had no effects on Pyk2 and ERK activations or the invasion of HBE cells, which suggested that in TrkB-positive A549 cells, up-regulated TrkB was readily activated upon BDNF, and signaling pathways initiated by TrkB led to an immediate activation of Pyk2 and Pyk2-mediated functions.

Recent studies have been shown that inactivation of Trk by tyrosine kinase inhibitors was correlated with the inhibited invasion of tumor cells [30] and aiming at interfering TrkB expression or activation might be helpful in the progression of effective anticancer therapies. Our TrkB knockdown experiments in this study demonstrated a critical role of TrkB in BDNF-induced Pyk2 and ERK activations and the invasion of A549 cells. Further investigations should be carried out for the detailed activation and interaction between TrkB and Pyk2 in other lung cancer cell lines or in vivo.

The involvement of Pyk2 in the invasion of TrkB-positive A549 cells was clearly evident that the phosphorylation in Tyr402 was up-regulated by BDNF as well as cell invasion. Since BDNF-induced cell invasion was significantly reduced in Pyk2 knockdown cells, it was indicated that Pyk2 was required for regulating the invasion of A549 cells. Pyk2-mediated functions were performed by activating multiple downstream signaling molecules, including ERK, p38, c-Src and paxillin, which led to the differential regulation of cell invasion in various cell types [31-34]. The activation of ERK was observed after BDNF treatment, which was inhibited by Pyk2-siRNA and concomitant with a decreased cell invasion. Thus, we considered that ERK activated by Pyk2 was participated in the invasion of A549 cells stimulated by BDNF. Further experiments are necessary to clarify if other signaling molecules are involved in BDNF-induced cell invasion.

Taken together, our study confirmed that TrkB was overexpressed in NSCLCs. When activated by BDNF, TrkB induced Pyk2 phosphorylation in Tyr402, which led to ERK activation and promoted cell invasion. Our data thus revealed a TrkB/Pyk2/ERK signaling pathway that regulated the invasion of A549 cells and provided potential targets for the metastasis of NSCLC. Nevertheless, other signaling pathway(s) involved in the TrkB-associated invasion of lung cancer cells required further studies.

## Conclusions

Our data suggested that TrkB was higher expressed in NSCLC and patients with more TrkB expression had significant metastatic lymph nodes. In A549 cells, when activated by BDNF, TrkB mediated Pyk2 phosphorylation in Tyr402, led to ERK activation and promoted cell invasion. Our data thus revealed the involvement of TrkB in lymph node metastasis of NSCLC and a TrkB/Pyk2/ERK signaling pathway that regulated the invasion of A549 cells and provided potential targets for the metastasis of NSCLC.

## Competing interests

The authors declare that they have no competing interests.

## Authors' contributions

SZ initiated the research, carried out the experiments and wrote the manuscript, DG contributed to the paper translation, WL and QZ gave experimental instructions, YZ, CL and YL helped with the experimental design, ZC and XQ gave funding support and critical review of the manuscript. All authors read and approved the final manuscript.

## Pre-publication history

The pre-publication history for this paper can be accessed here:

http://www.biomedcentral.com/1471-2407/10/43/prepub
